# Long-Term Recurrence After Transanal Hemorrhoidal Dearterialization Versus Excisional and Stapled Surgery for Hemorrhoidal Disease: A Systematic Review and Meta-Analysis of Studies with ≥24 Months of Follow-Up

**DOI:** 10.3390/jcm15197606

**Published:** 2026-09-30

**Authors:** Moaz W. Abulfaraj, Ghader Jamjoom, Sultan Almuallem, Ghada H. Postaji, Taibeh S. Alamoudi, Doaa A. Halawani, Juri M. Alhazmi, Mrwan M. Almutairi, Murad Aljiffry

**Affiliations:** 1 Department of Surgery, Faculty of Medicine, King Abdulaziz University, Jeddah 21589, Saudi Arabia; smalmuallem@kau.edu.sa (S.A.); maljefri@kau.edu.sa (M.A.); 2 Department of General Surgery, King Abdulaziz University Hospital, King Abdulaziz University, Jeddah 21589, Saudi Arabia; ggjamjoom@kau.edu.sa; 3 College of Medicine, Ibn Sina National College for Medical Studies, Jeddah 22421, Saudi Arabia; 2120376@ibnsina.edu.sa (G.H.P.); 1721024@ibnsina.edu.sa (T.S.A.); 2120246@ibnsina.edu.sa (J.M.A.); 4 College of Medicine, King Saud bin Abdulaziz University for Health Sciences, Jeddah 21423, Saudi Arabia; halawani20071@ksau-hs.edu.sa; 5 Faculty of Medical Sciences, University of Groningen, 9713 GZ Groningen, The Netherlands; mrwan.m.almutairi@gmail.com

**Keywords:** hemorrhoidal disease, transanal hemorrhoidal dearterialization, hemorrhoidal artery ligation, hemorrhoidectomy, stapled hemorrhoidopexy, recurrence, long-term outcomes, systematic review, meta-analysis

## Abstract

**Background/Objectives**: Hemorrhoidal dearterialization offers less postoperative pain and faster recovery than excisional hemorrhoidectomy, but prior meta-analyses pooled recurrence at predominantly 6–12-month follow-up; long-term durability has never been quantified. We compared recurrence at ≥24 months after dearterialization versus excisional hemorrhoidectomy and stapled hemorrhoidopexy or anopexy. **Methods**: Five databases were searched up to 19 August 2026 (PROSPERO CRD420261362253). Randomized trials and comparative cohorts of dearterialization reporting recurrence with ≥24 months of follow-up in both arms were eligible. Screening, extraction, and risk-of-bias assessment were performed in duplicate; random-effects risk ratios (RR) were pooled separately by study design and comparator class, with Hartung–Knapp and continuity-correction sensitivity analyses. **Results**: Thirteen studies were included (6 randomized trials, 7 cohorts; 3283 patients). Versus excisional hemorrhoidectomy, recurrence was higher after dearterialization in the 4 randomized trials (241 patients, 12 events; RR 3.67, 95% CI 1.04–12.97) and in the 5 cohorts (RR 1.71, 95% CI 1.31–2.21); the combined estimate, weighted toward the cohorts, was RR 1.76 (95% CI 1.36–2.27; crude recurrence 12.1% vs. 6.5%). Versus stapled techniques (4 studies, 521 patients), the difference was not significant (RR 2.07, 95% CI 0.89–4.81). Recurrences after dearterialization clustered in grade IV disease (21.5% vs. 4.2% for grade III). Dearterialization had fewer overall complications (RR 0.76, 95% CI 0.60–0.95) and fewer late complications (RR 0.19, 95% CI 0.04–0.85; 9 events). **Conclusions**: At two or more years, dearterialization is associated with higher recurrence than excisional hemorrhoidectomy; the randomized evidence is imprecise, and the combined estimate is supportive rather than definitive. Recurrence after dearterialization clustered in grade IV disease, and late morbidity may be lower; both findings are descriptive. Long-term evidence versus stapled techniques remains limited. These findings may help inform patient counseling on the trade-off between durability and largely irreversible harms.

## 1. Introduction

Hemorrhoidal disease affects a substantial proportion of the adult population, and surgery remains the most effective treatment for grade III–IV disease [1,2]. Excisional hemorrhoidectomy (Milligan–Morgan, Ferguson, and vessel-sealing variants) provides durable results but is associated with significant postoperative pain and complications including bleeding, chronic wounds, anal stenosis, and impaired continence [3,4]. Transanal hemorrhoidal dearterialization (THD) and Doppler-guided hemorrhoidal artery ligation (DG-HAL), usually combined with mucopexy or recto-anal repair (RAR), avoid anodermal wounds and sphincter-adjacent excision. Early pain is lower and recovery faster [5,6].

Multiple randomized trials and meta-analyses have compared dearterialization with excisional and stapled surgery [7,8,9,10]. However, these syntheses share a limitation: recurrence was pooled at whatever follow-up each study reported, predominantly 6–12 months. The most recent pairwise meta-analysis (Jee et al., 2024) included six RCTs searched to December 2022, pooled recurrence across mixed short follow-up, and performed no stratification by follow-up duration [10]. A 2023 network meta-analysis of surgical procedures did not include dearterialization at all [11], and current guidelines still differ on the place of non-excisional techniques in grade III disease [2,12]. A bibliometric analysis of two decades of hemorrhoid research likewise identified long-term comparative effectiveness as a persisting knowledge gap [13]. Because the failure mode of dearterialization is late prolapse recurrence, short-horizon pooling may systematically favor the technique. It also leaves unanswered the question that matters most when counseling patients: whether the early advantages are preserved at 2–5 years. Recent trial reports have called for long-term synthesis [14,15], and the long-term comparative evidence base has grown substantially since 2022, including a 1681-patient multicenter study with 24-month follow-up (EMODART3) [15], a multicenter prospective comparison at a median of 3 years [16], and the 2-year report of the THDLIGA randomized trial [14]. A randomized trial published in 2025 also found substantially higher clinical failure after THD than after open excision within the first year [17], which makes the durability question beyond that horizon all the more pressing.

We therefore conducted a systematic review and meta-analysis restricted to comparative studies reporting recurrence at ≥24 months after dearterialization versus excisional hemorrhoidectomy or stapled hemorrhoidopexy/anopexy, with pre-specified analyses by hemorrhoid grade, mucopexy, follow-up stratum, and recurrence definition.

## 2. Materials and Methods

### 2.1. Protocol and Registration

The review was conducted according to PRISMA 2020 [18] and registered on PROSPERO (CRD420261362253) before formal data extraction. The protocol narrowed an earlier broad-scope draft to the long-term question; all amendments were made before formal screening and are documented in the registration. The PRISMA 2020 checklist is provided in the Supplementary Materials as Table S1.

### 2.2. Eligibility Criteria

We included randomized controlled trials and comparative cohort studies (prospective or retrospective, including propensity-matched analyses) of adults (≥18 years) with grade II–IV hemorrhoidal disease comparing dearterialization in any variant (THD, DG-HAL, HAL-RAR, non-Doppler HAL, with or without mucopexy) against excisional hemorrhoidectomy (Milligan–Morgan, Ferguson, diathermy, vessel-sealing) or stapled hemorrhoidopexy/anopexy including tissue-selecting therapy (TST). The primary outcome was recurrence (symptomatic, examination-confirmed, or reintervention, as defined by each study) reported with ≥24 months of follow-up (mean, median, or fixed timepoint) in both the dearterialization and comparator arms; follow-up schedules were required to be the same for both arms. Studies with follow-up below this threshold, single-arm series, dearterialization-variant comparisons, and rubber band ligation comparators were excluded. No language restriction was applied.

### 2.3. Search Strategy and Study Selection

**Figure 1:**
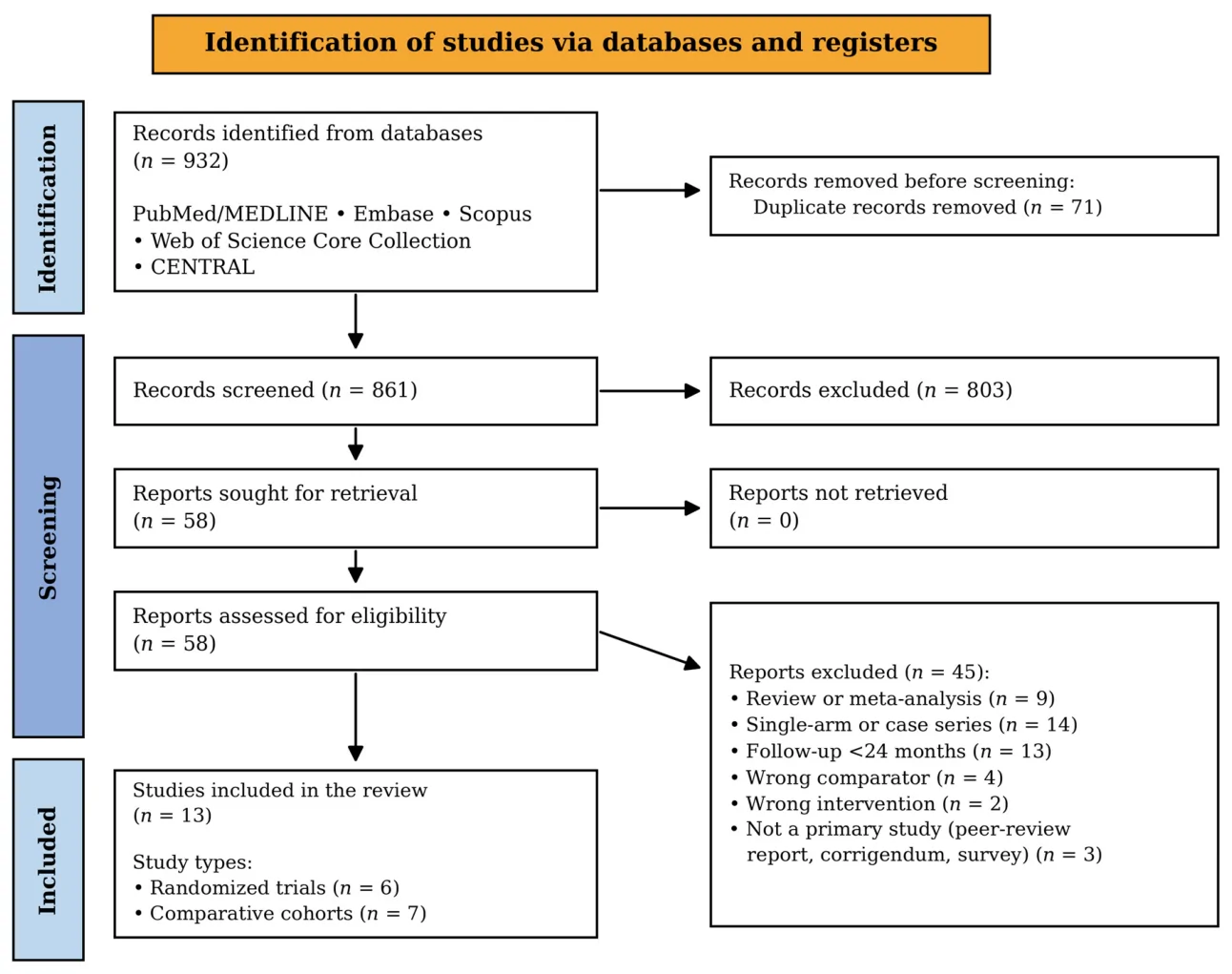
PRISMA 2020 flow diagram of study identification and selection.

PubMed/MEDLINE, Embase, Scopus, Web of Science Core Collection, and CENTRAL were searched from inception to 19 August 2026 using intervention and comparator terms without outcome terms (full strategies in Supplementary Text S1). Supplementary sources included ClinicalTrials.gov, WHO ICTRP, Google Scholar, and backward/forward citation chasing of included studies and prior reviews. Three reviewers (G.H.P., T.S.A., G.J.) independently executed the searches in all databases; results were merged and deduplicated. Two reviewers (J.M.A., D.A.H.) independently screened titles/abstracts and full texts, with disagreements resolved by a third reviewer. Reasons for full-text exclusion were documented (PRISMA flow diagram, Figure 1).

### 2.4. Data Extraction and Risk of Bias

Two reviewers independently extracted study characteristics, intervention details (device, Doppler use, mucopexy), comparator technique, follow-up duration and ascertainment method, recurrence definition, raw event counts and denominators at final follow-up (and per grade where reported), reinterventions, losses to follow-up, and complications. Authors were contacted where ≥24-month data were reported without extractable counts. Risk of bias was assessed with RoB 2 for randomized trials and ROBINS-I for non-randomized studies; studies described as “casually allocated” without a described randomization method were treated as non-randomized.

### 2.5. Statistical Analysis

Risk ratios with 95% CIs were pooled with DerSimonian–Laird random-effects models, separately for each comparator class; RCTs and non-randomized studies were pooled separately, with combined estimates presented as secondary, supportive analyses. Because DerSimonian–Laird intervals can be too narrow with few studies, every pooled estimate was re-estimated with the Hartung–Knapp adjustment (truncated to be no narrower than the standard interval). Zero-cell studies received 0.5 continuity corrections; the influence of this choice was examined with 0.1 and 1.0 corrections, by excluding zero-event studies, and with Peto odds ratios; late-complication analyses were also repeated with risk differences. Heterogeneity was quantified with I^2^ and τ^2^; prediction intervals are reported where ≥3 studies contributed. Because I^2^ has little power with few studies and sparse events, absence of statistical heterogeneity was not interpreted as clinical homogeneity. Recurrence was analyzed as reported by each study (any recurrence); reintervention was additionally synthesized as a stand-alone outcome where reported, and crude absolute recurrence proportions are presented. Pre-specified subgroups: grade III vs. IV; mucopexy vs. none; follow-up 24–36 vs. >36 months; recurrence definition. Sensitivity analyses: ≥36-month follow-up; RCT-only; low/moderate risk of bias; ≥18-month threshold; leave-one-out. Small-study effects were assessed by funnel plot and Egger test where ≥10 studies. Certainty was rated with GRADE. Analyses were performed in Python 3.12 (Python Software Foundation, Wilmington, DE, USA) with NumPy 2.4 and SciPy 1.17, and figures were produced with Matplotlib 3.10. Analysis code is available from the corresponding author.

## 3. Results

### 3.1. Study Selection and Characteristics

Of 932 records identified across the five databases, 71 duplicates were removed and 861 records were screened; 803 were excluded at title/abstract, 58 reports were sought and all 58 full texts were assessed; 13 studies met eligibility criteria: 6 randomized trials [14,19,20,21,22,23] and 7 comparative cohorts [15,16,24,25,26,27,28], totaling 3283 patients with follow-up from 24 months to a mean of 7 years (Table 1; Figure 1). Nine studies compared dearterialization with excisional hemorrhoidectomy (2762 patients) and four with stapled hemorrhoidopexy, anopexy, or TST (521 patients); two cohorts included small proportions of grade II disease. Mucopexy was performed in all dearterialization arms where reported. Follow-up duration and schedule were the same for both arms in every included study. Lauricella et al. was excluded from the primary analysis because its defined recurrence endpoint was ascertained before 24 months [29].

### 3.2. Risk of Bias

Four of the six randomized trials were rated as having some concerns, chiefly for undescribed allocation concealment and unblinded outcome assessment, and two at high risk of bias (32.5% attrition at 3 years in one; undescribed randomization with post-randomization exclusions in the other). Two of the seven non-randomized studies were rated at moderate and five at serious risk on ROBINS-I, chiefly for confounding by treatment selection and for outcome ascertainment. Study-level ratings are given in Table 1 and Figures S2 and S3.

### 3.3. Long-Term Recurrence Versus Excisional Hemorrhoidectomy

**Figure 2:**
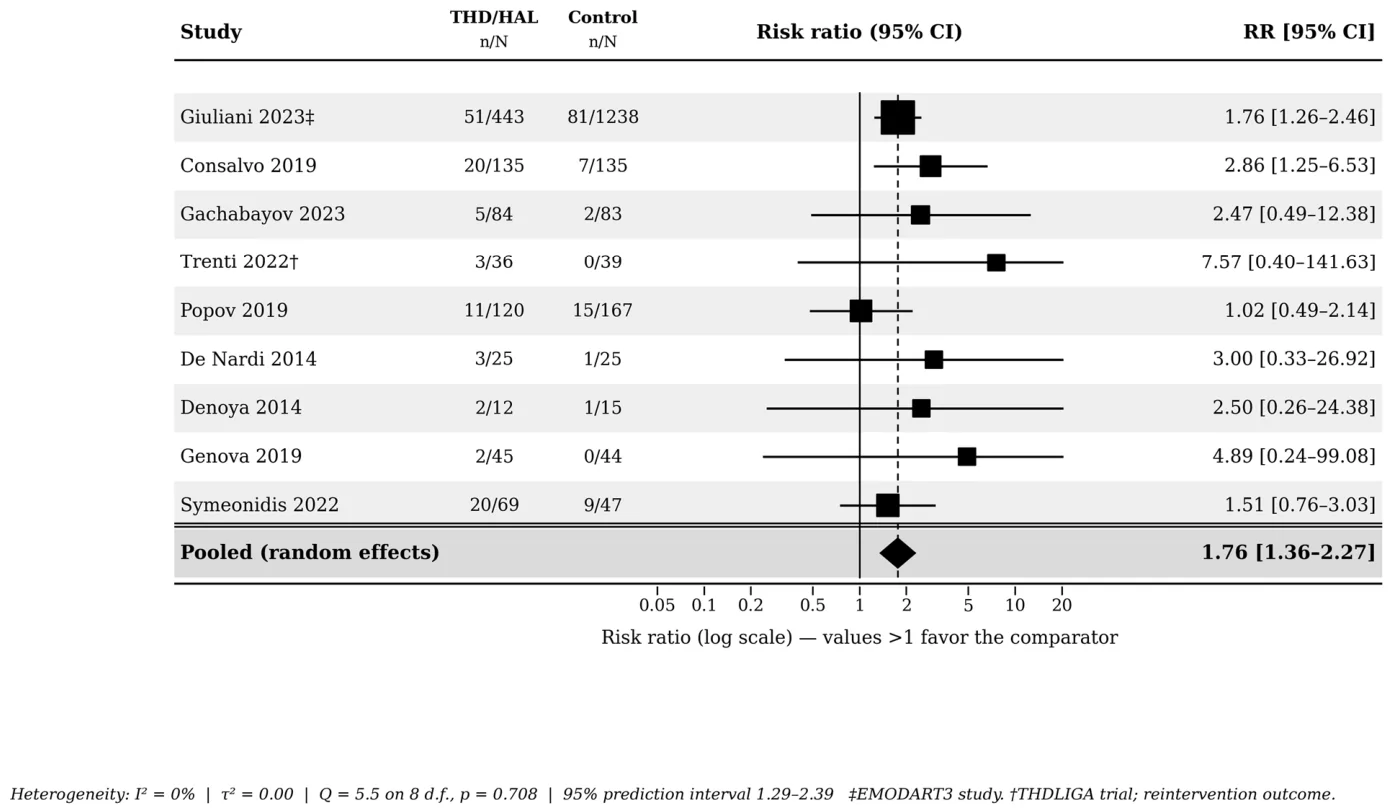
Forest plot of recurrence at ≥24 months: dearterialization versus excisional hemorrhoidectomy (9 studies) [14,15,16,19,20,21,24,25,28].

Among randomized trials (4 trials, 241 patients, 12 events), recurrence at ≥24 months was higher after dearterialization: RR 3.67 (95% CI 1.04–12.97; Hartung–Knapp 95% CI 0.47–28.51, *p* = 0.14; I^2^ = 0%; Figure S1). The estimate was also sensitive to the handling of zero-event trials: RR 3.54 with a 0.1 correction, 3.15 with a 1.0 correction, and 2.75 (95% CI 0.57–13.34) after excluding the two trials with no comparator events. The Peto odds ratio was 4.40 (95% CI 1.38–14.06). Among non-randomized studies (5 studies, 2521 patients), the estimate was concordant and more precise (RR 1.71, 95% CI 1.31–2.21; Hartung–Knapp 1.18–2.47; I^2^ = 0%). The pre-specified secondary synthesis combining all 9 studies (2762 patients; 117 events after dearterialization vs. 116 after excision) gave RR 1.76 (95% CI 1.36–2.27; Hartung–Knapp 1.30–2.37; I^2^ = 0%; 95% prediction interval 1.29–2.39; Figure 2), corresponding to crude absolute recurrence of 12.1% (117/969) after dearterialization versus 6.5% (116/1793) after excision. The direction of effect was consistent in 8 of 9 studies. The combined estimate was essentially unchanged on leave-one-out analysis, including after removal of the largest study (EMODART3, 52% weight: RR 1.76, 95% CI 1.18–2.61); after exclusion of the one trial contributing reintervention rather than symptomatic recurrence (THDLIGA: RR 1.74, 95% CI 1.35–2.25); and after exclusion of all zero-event studies (RR 1.73, 95% CI 1.34–2.23).

### 3.4. Long-Term Recurrence Versus Stapled Techniques

**Figure 3:**
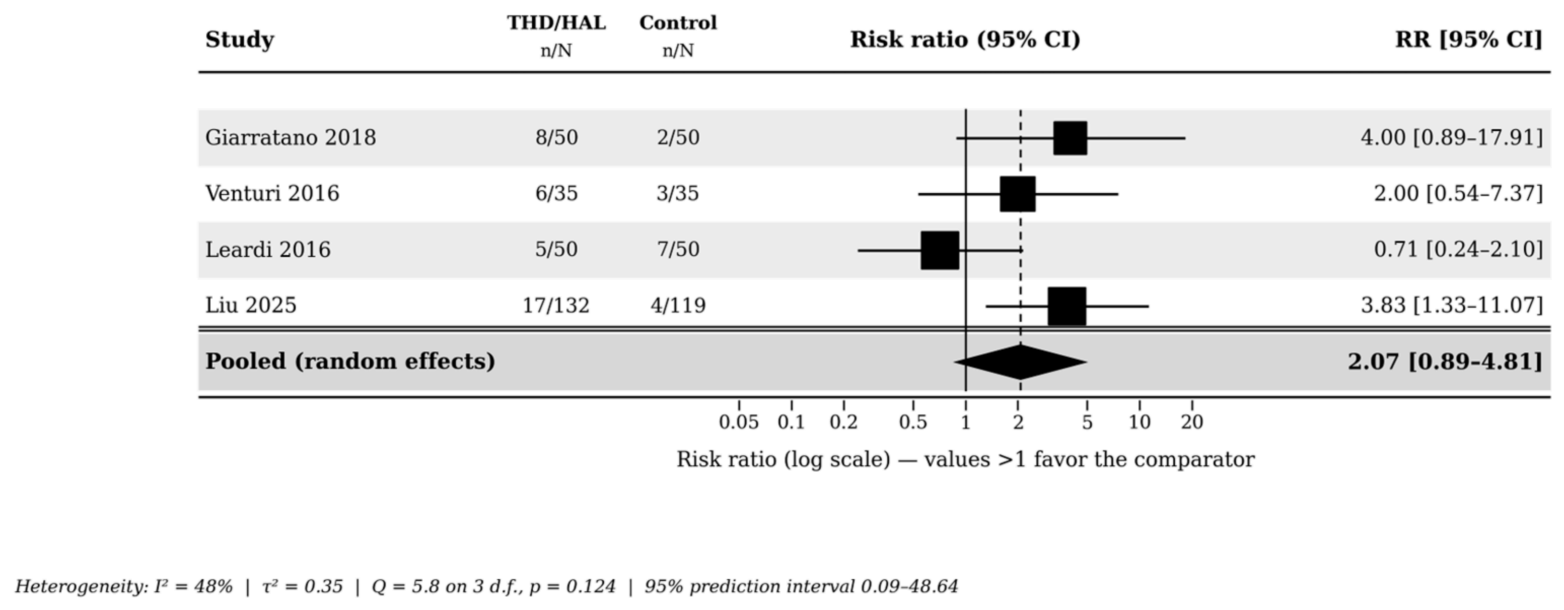
Forest plot of recurrence at ≥24 months: dearterialization versus stapled hemorrhoidopexy/anopexy/TST (4 studies) [22,23,26,27].

Across 4 studies (521 patients), the pooled estimate favored stapled hemorrhoidopexy/anopexy/TST without reaching significance (RR 2.07, 95% CI 0.89–4.81; Hartung–Knapp 0.52–8.14; I^2^ = 48%) (Figure 3). The two randomized trials alone [22,23] gave RR 2.70 (95% CI 1.01–7.21; 22 events). With four studies of different stapled techniques, this comparison supports neither equivalence nor superiority. Heterogeneity was driven by the study with the longest follow-up (mean 7 years), which favored dearterialization [26].

### 3.5. Hemorrhoid Grade

**Figure 4:**
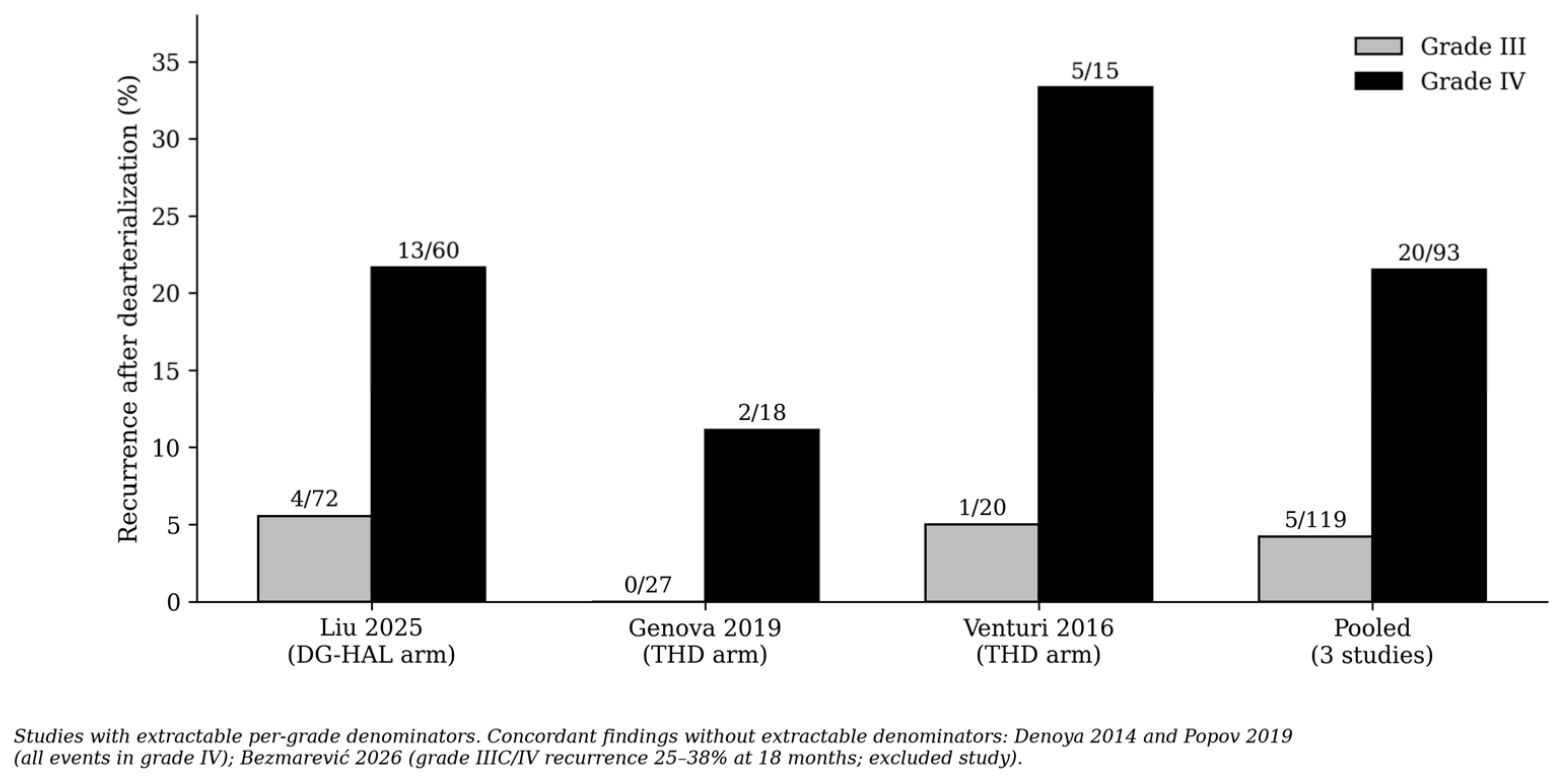
Recurrence after dearterialization by hemorrhoid grade in studies with extractable per-grade denominators [21,23,27].

In every study reporting grade-level outcomes, long-term recurrences clustered in grade IV disease: 21.7% vs. 5.6% (grade IV vs. III) after DG-HAL [27]; all recurrences in grade IV strata in two randomized trials [20,21]; 33.3% vs. 5.0% (grade IV vs. III) after THD in a third randomized trial [23]; and all reoperations after dearterialization occurring in grade IV patients in the largest prospective cohort [24]. Pooled dearterialization-arm recurrence in the three studies with extractable per-grade denominators was 21.5% (20/93) for grade IV versus 4.2% (5/119) for grade III (Figure 4). In the two randomized trials with comparative per-grade data [21,23], the grade IV comparison favored the comparator (RR 2.90, 95% CI 0.77–10.86) whereas the grade III comparison showed no difference (RR 0.98, 95% CI 0.11–8.97); a formal test of interaction between grade and treatment effect was not estimable.

### 3.6. Complications

**Figure 5:**
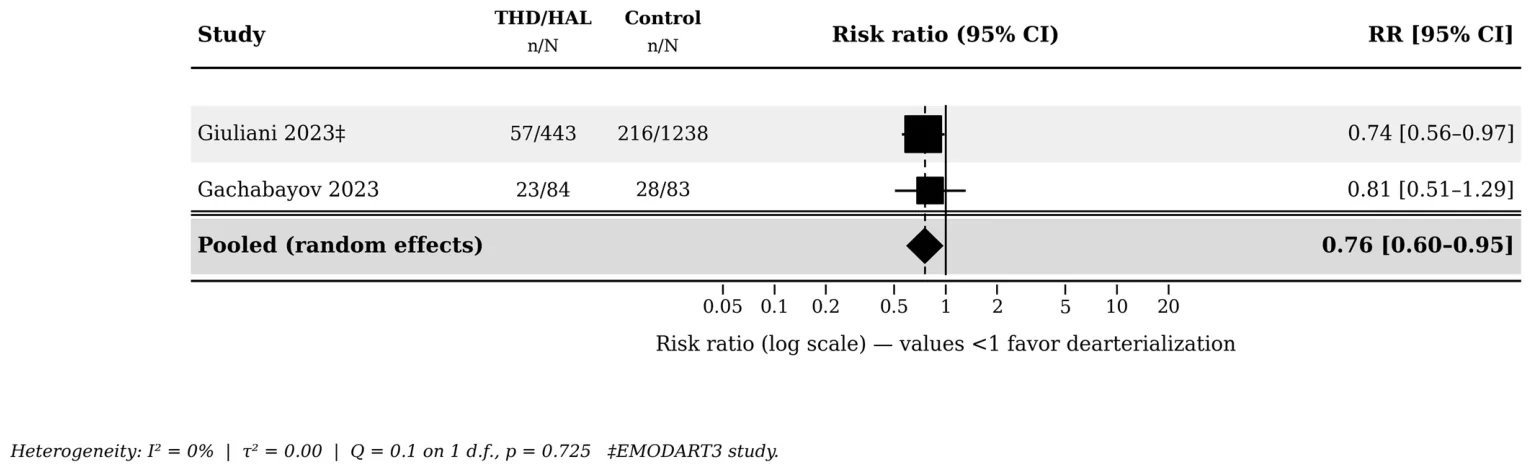
Forest plot of postoperative complications of any type: dearterialization versus excisional hemorrhoidectomy [15,16].

**Figure 6:**
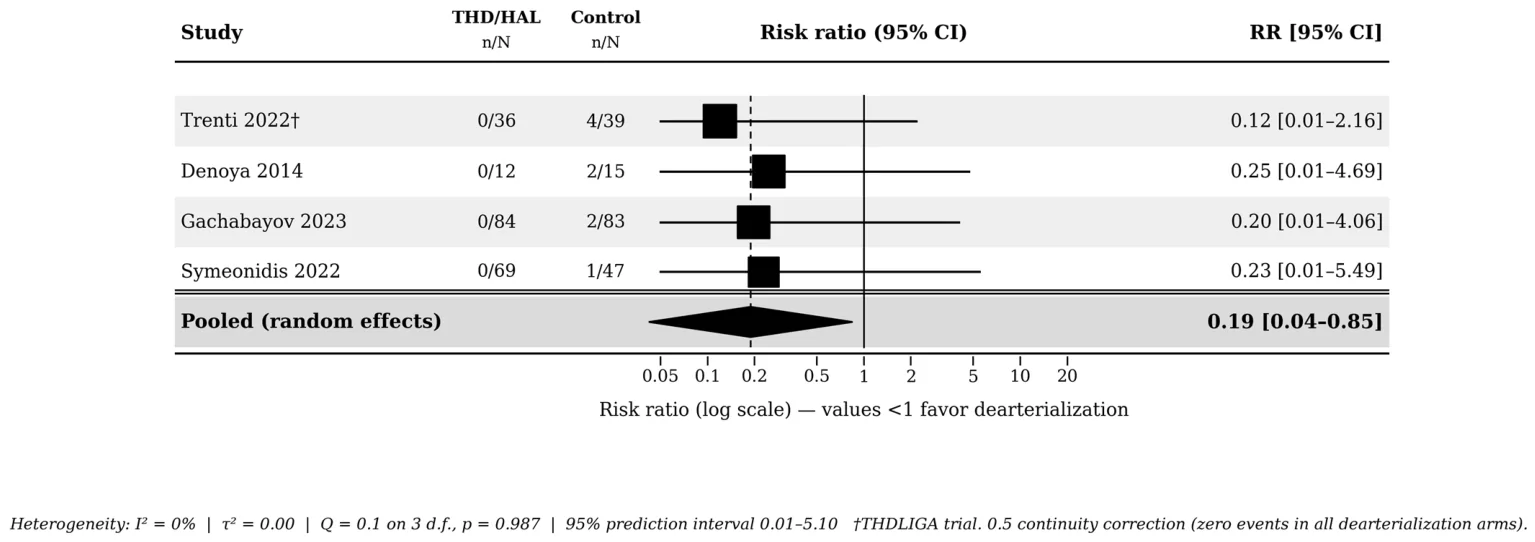
Forest plot of late complications (anal stenosis, chronic wound, incontinence): dearterialization versus excisional surgery [14,16,20,25].

Dearterialization was associated with fewer postoperative complications of any type, as defined by each study (2 studies, 1848 patients: RR 0.76, 95% CI 0.60–0.95; *p* = 0.019; I^2^ = 0%; Figure 5); the complication windows differed between the two studies (30-day Clavien–Dindo vs. whole follow-up). Late complications (anal stenosis, chronic open wound, fecal incontinence) were reported in 0 patients after dearterialization versus 9 after excisional or vessel-sealing surgery across the four reporting studies (RR 0.19, 95% CI 0.04–0.85; Peto OR 0.13, 95% CI 0.03–0.50; pooled risk difference −3.5%, 95% CI −6.6% to −0.3%; Figure 6). With a 0.1 continuity correction, the RR was 0.05 (95% CI 0.00–1.09). No sphincter lesions were recorded after dearterialization in any study performing endoanal ultrasonography [14,16], and no late complications occurred in either arm of the randomized comparison with stapled anopexy [23].

### 3.7. Secondary Outcomes and Additional Analyses

By follow-up stratum, the excisional comparison gave consistent estimates at 24–36 months (4 studies: RR 1.83, 95% CI 1.32–2.52) and beyond 36 months (5 studies: RR 1.66, 95% CI 1.09–2.54; I^2^ = 3%). Restricting to studies at low or moderate risk of bias (4 studies) gave RR 1.82 (95% CI 1.00–3.32; *p* = 0.049). By recurrence definition, the effect was present in studies using symptomatic or patient-reported definitions (3 studies: RR 1.59, 95% CI 1.20–2.10) and larger in studies using examination-confirmed definitions (4 studies: RR 2.92, 95% CI 1.43–5.95). Reintervention as a stand-alone outcome was reported by three studies versus excisional surgery (THDLIGA 3/36 vs. 0/39; De Nardi 1/25 vs. 1/25; Popov 3/120 vs. 5/167): crude 3.9% versus 2.6%, RR 1.21 (95% CI 0.38–3.84; 13 events). In the studies reporting both outcomes, the proportion of recurrences that led to reoperation varied (3 of 11 in Popov et al. [24]; 5 of 6 in Venturi et al. [23]). Two pre-specified subgroup analyses could not be performed: mucopexy versus none (mucopexy was performed in all dearterialization arms where reported) and comparator subtype within the excisional class (strata too sparse). The pre-specified ≥18-month sensitivity analysis added no study with extractable comparative counts and was not performed. Funnel-plot and Egger-test assessment was not performed because no comparison reached the pre-specified threshold of 10 studies. Certainty of evidence (GRADE) was low for recurrence versus excisional hemorrhoidectomy and for both complication outcomes, and very low versus stapled techniques (Summary of Findings, Table 2).

## 4. Discussion

This is the first systematic review to synthesize comparative outcomes of hemorrhoidal dearterialization restricted to ≥24 months of follow-up. Three findings emerge. First, dearterialization was associated with higher long-term recurrence than excisional hemorrhoidectomy in both randomized and observational studies, with the same direction in 8 of 9 studies. Because most of the pooled weight comes from observational studies, the combined RR of 1.76 supports, rather than establishes, an approximately 76% higher relative recurrence risk. In absolute terms, this corresponds to roughly 12% versus 7% across the pooled studies, and to approximately 6–12% versus 2–7% in the three grade III-restricted studies. In the 2025 randomized trial noted above, all eight reoperations within the first year occurred in the THD arm [17]. Second, recurrences after dearterialization clustered in grade IV disease in every study reporting grade-level data. This pattern is consistent but descriptive, and it should be regarded as hypothesis-generating rather than as a basis for grade-specific treatment recommendations. Third, the trade-off is two-sided. Dearterialization had fewer overall complications, and no anal stenosis, chronic wound, or incontinence was reported in its arms in the four studies reporting late complications. Recurrence and permanent morbidity are also not equivalent outcomes: in the studies reporting both, the proportion of recurrences after dearterialization that led to reoperation ranged from 3 of 11 to 5 of 6, and pooled reintervention did not differ significantly between techniques, whereas sphincter injury and stenosis are largely irreversible. Informed consent should present both sides.

The grade gradient is consistent with large single-arm series, in which recurrence after THD was 18.1% in grade IV versus 8.7% in grade III over a mean of 44 months [30], and 31.3% versus 8.2% after DG-HAL at one year [31]. The same durability gradient is seen with other non-excisional techniques: in 686 patients with grade III disease, two-year recurrence was 14.5% after suture mucopexy alone versus 4.2% after Milligan–Morgan hemorrhoidectomy [32], and in a randomized trial with 5-year follow-up, symptom recurrence was 44% after sutured mucopexy versus 24% after excision [33]. Doppler guidance itself does not appear to reduce recurrence [34]. Durability may therefore depend more on how prolapse is corrected than on arterial ligation. The favorable late-morbidity profile is supported by a recent systematic review of continence after artery ligation, which found low-grade incontinence in 2.5% of 671 patients and no severe cases [35].

Our findings help explain the prior literature. Meta-analyses pooling predominantly 12-month data reported inconsistent or attenuated recurrence differences [8,9,10]; our restriction to ≥24 months clarifies this inconsistency and indicates a durable difference versus excision. The comparison against stapled techniques (four heterogeneous studies) remains inconclusive and should not be used to guide technique selection in grade IV disease; the divergent 7-year cohort suggests the two prolapse-correcting approaches may converge over very long horizons, a hypothesis that remains untested. Heterogeneity in recurrence definitions, with patient-reported rates exceeding examination-confirmed rates several-fold in the same cohorts [16,21], emerged as a major explanation for the variability of the prior literature.

### 4.1. Limitations

Several limitations qualify these findings. Seven of thirteen included studies were non-randomized, and the combined estimate is weighted toward cohorts at serious risk of bias, so GRADE certainty is low. The randomized subset comprises 241 patients and 12 events, and its precision depends on the estimator: significant with DerSimonian–Laird and Peto methods but not with the Hartung–Knapp adjustment. The absence of statistical heterogeneity should not be read as clinical homogeneity: the studies differed in technique, grade mix, recurrence definition, and follow-up method, and with few studies and sparse events, I^2^ has little power to detect such variation. One large study contributed approximately half of the weight in the combined analysis, although the pooled estimate was identical after its removal. Recurrence definitions varied across studies, and reintervention could be synthesized from only three studies. Complication reporting was incomplete, and the late-complication estimate rests on nine events with zero cells; the GRADE wording “may reduce” reflects this. The grade analysis is descriptive, based on three studies, and no test of interaction was possible. The search strategy included study-design terms, so comparative studies not indexed with those terms could theoretically have been missed, although citation chasing of all included studies and prior reviews mitigates this.

### 4.2. Future Directions

Grade-stratified randomized trials with at least three years of follow-up in both arms are needed to test whether grade modifies the relative durability of dearterialization. Outcomes should be reported with validated patient-reported instruments such as the Hemorrhoidal Disease Symptom Score [36], with examination-confirmed recurrence and reintervention reported separately. Individual patient data meta-analysis or prospective registries could supply the grade-level data needed for a formal interaction analysis, and an ongoing network meta-analysis of randomized trials that plans to stratify by follow-up duration [37] should help place the non-randomized evidence in context. Dedicated long-term comparisons against stapled and laser techniques, with cost-effectiveness analyses, remain a priority.

## 5. Conclusions

At two or more years of follow-up, hemorrhoidal dearterialization is associated with higher recurrence than excisional hemorrhoidectomy; the randomized evidence is imprecise, and the combined estimate is supportive rather than definitive. Recurrences after dearterialization clustered in grade IV disease, and dearterialization may reduce overall and late morbidity. Long-term evidence versus stapled techniques remains limited. These findings may help inform patient counseling on the trade-off between durability and largely irreversible harms.

## Figures and Tables

**Table 1:** Characteristics of included studies.

Study (Country)	Design	Comparison (I vs. C)	n (I/C)	Grade	Mucopexy	Follow-Up	Recurrence Definition	Recurrence I vs. C	Risk of Bias
Giuliani 2023 [15], EMODART3 (Italy/UK)	Multicenter retrospective (28 centers); matched subanalysis	HAL/THD + mucopexy vs. excisional (MM; diathermy/LigaSure/Harmonic)	443/1238	III only	Yes (all)	24 mo (fixed)	Symptom score ≥ preop (58% telephone)	11.5% (51/443) vs. 6.5% (81/1238)	Serious (ROBINS-I) ^1^
Consalvo 2019 [28] (Italy)	Retrospective comparative	THD + Doppler arterial identification vs. classic hemorrhoidectomy	135/135	III–IV	Yes	3 y	Clinical examination (records)	14.8% (20/135) vs. 5.2% (7/135)	Serious (ROBINS-I) ^2^
Gachabayov 2023 [16] (USA, multicenter)	Prospective non-randomized, video-audited	THD + mucopexy vs. Ferguson	84/83	Prolapsing reducible, ≥3 columns (≈III)	Yes	Median 3.0 y (1.0–5.5)	Office visit + patient-reported	5.9% (5/84) vs. 2.4% (2/83)	Moderate (ROBINS-I)
Trenti 2022 [14], THDLIGA (Spain)	Multicenter RCT (2-year report)	THD + mucopexy vs. vessel-sealing hemorrhoidectomy	36/39	III–IV	Yes	2 y (fixed)	Symptom score; reintervention	Reintervention 8.3% (3/36) vs. 0% (0/39)	Some concerns (RoB 2)
Popov 2019 [24] (Bulgaria)	Prospective comparative	DG-THD vs. conventional hemorrhoidectomy	120/167	II–IV	NR/partial	Mean 46 ± 16 mo (18–78)	Symptomatic recurrence	9.2% (11/120) vs. 9.0% (15/167)	Serious (ROBINS-I) ^2^
De Nardi 2014 [19] (Italy)	RCT	DG-THD + mucopexy vs. open hemorrhoidectomy	25/25	III	Yes	24 mo (fixed)	Symptoms + examination; reintervention	12% (3/25) vs. 4% (1/25)	Some concerns (RoB 2)
Denoya 2014 [20] (USA)	Double-blind RCT (3-year extension)	THD + mucopexy vs. hemorrhoidectomy	12/15 analyzed (20/20 randomized)	III–IV	Yes	Median 35 mo (27–43)	Proctoscopy-confirmed	16.7% (2/12) vs. 6.7% (1/15)	High (RoB 2) ^3^
Genova 2019 [21] (Italy)	RCT (method not described)	THD + mucopexy vs. Milligan–Morgan	45/44	III (27/25) + IV (18/19)	Yes	3 y (rectoscopy)	Clinical + rectoscopy	4.4% (2/45) vs. 0% (0/44)	High (RoB 2) ^4^
Symeonidis 2022 [25] (Greece)	Retrospective, clinic-based allocation	HAL-RAR vs. Milligan–Morgan	69/47	II/III/IV: 6/35/28 vs. 9/22/16	Yes (RAR)	Median 41 mo (min 24–72)	Telephone interview, symptomatic	29.0% (20/69) vs. 19.1% (9/47)	Moderate (ROBINS-I) ^5^
Giarratano 2018 [22] (Italy)	RCT	THD vs. stapled hemorrhoidopexy	50/50	III–IV	NR	Mean 33.7 ± 7.6 mo	Clinical examination	16% (8/50) vs. 4% (2/50)	Some concerns (RoB 2)
Venturi 2016 [23] (Italy)	RCT, grade-stratified (permuted blocks, sealed envelopes)	THD + anopexy vs. stapled anopexy	35/35 (III: 20/20; IV: 15/15)	III–IV	Yes (anopexy)	Mean 36 ± 6 mo (to 42)	Residual prolapse at clinical examination	17.1% (6/35) vs. 8.6% (3/35); grade IV 33.3% vs. 13.3%	Some concerns (RoB 2)
Leardi 2016 [26] (Italy)	Comparative, non-randomized	DG-THD vs. stapled hemorrhoidopexy	50/50	III	NR	Mean 7.0 y (5–11)	Examination; persistence/recurrence	10% (5/50) vs. 14% (7/50)	Serious (ROBINS-I) ^6^
Liu 2025 [27] (China)	Retrospective cohort	DG-HAL vs. TST	132/119	III–IV	NR	3 y (telephone/outpatient)	Prolapse recurrence (bleeding separate)	12.9% (17/132) vs. 3.4% (4/119)	Serious (ROBINS-I) ^2^

I = dearterialization arm (THD/DG-HAL/HAL-RAR); C = comparator; NR = not reported. ^1^ Inclusion required 24-month follow-up availability; self-reported multicenter data (mitigated by regression adjustment and matched case–control subanalysis). ^2^ Treatment allocation opaque/by surgeon choice. ^3^ 32.5% attrition at 3 years. ^4^ Randomization method not described; 9 refusers excluded after selection. ^5^ Quasi-random allocation by outpatient clinic. ^6^ Described as “casually allocated” without a stated method; treated as non-randomized. Lauricella 2024 [29]: excluded from primary analysis (recurrence endpoint < 24 months). LigaSure (Medtronic, Minneapolis, MN, USA); Harmonic (Ethicon, Johnson & Johnson, Cincinnati, OH, USA).

**Table 2:** GRADE Summary of Findings.

Outcome (Comparison)	Studies/Patients	Design Mix	Effect Estimate (95% CI)	Downgrades	Certainty	Plain-Language Statement
Recurrence ≥ 24 mo (vs. excisional hemorrhoidectomy)	9/2762	RCT + NRS; NRS-weight dominant	RR 1.76 (1.36–2.27); HK 1.30–2.37; PI 1.29–2.39	Risk of bias (−1: serious in weight-dominant studies)	Low	Dearterialization may increase long-term recurrence; estimate weighted toward cohorts at serious risk of bias and supportive rather than definitive
Recurrence ≥ 24 mo, RCT-only sensitivity (vs. excisional)	4/241	RCTs only	RR 3.67 (1.04–12.97); HK 0.47–28.51; Peto OR 4.40 (1.38–14.06)	Risk of bias (−1); imprecision (−1: 12 events)	Low	Randomized evidence is directionally concordant but imprecise (12 events); precision depends on the estimator
Recurrence ≥ 24 mo (vs. stapled hemorrhoidopexy/anopexy/TST)	4/521	2 RCT + 2 NRS	RR 2.07 (0.89–4.81); HK 0.52–8.14	Risk of bias (−1); inconsistency (−1: I^2^ = 48%); imprecision (−1)	Very low	Evidence is insufficient to compare long-term recurrence between the two prolapse-correcting approaches
Overall postoperative complications (vs. excisional)	2/1848	2 NRS	RR 0.76 (0.60–0.95)	Risk of bias (−1); indirectness (−1: mixed time windows)	Low	Dearterialization may reduce overall postoperative complications
Late complications: stenosis, chronic wound, incontinence (vs. excisional)	4/385	2 RCT + 2 NRS	RR 0.19 (0.04–0.85); Peto OR 0.13 (0.03–0.50); RD −3.5% (−6.6 to −0.3)	Imprecision (−1: 9 events, zero cells)	Low	Dearterialization may reduce late morbidity; no events were reported after dearterialization in the four reporting studies, but the estimate rests on nine events

RR = risk ratio; PI = 95% prediction interval; RD = risk difference; NRS = non-randomized studies. GRADE ratings assessed by two reviewers. Bodies of evidence combining randomized and non-randomized designs start at the level of the design contributing most weight.

## Data Availability

The data supporting the findings of this study are contained within the article and its Supplementary Materials: study-level event counts, denominators, follow-up, recurrence definitions, and risk-of-bias ratings are reported in Table 1 and Figure 2, Figure 3, Figure 4, Figure 5, Figure 6 and Figures S1–S3, and the full search strategies in Text S1. Analysis code is available from the corresponding author on request. The review protocol is registered on PROSPERO (CRD420261362253).

## Supplementary Materials

The following supporting information can be downloaded at: https://www.mdpi.com/article/10.3390/jcm15197606/s1, Supplementary Text S1: Full search strategies; Figure S1: Sensitivity analysis restricted to randomized trials: recurrence at ≥24 months, dearterialization versus excisional hemorrhoidectomy (4 trials); Figure S2: Risk-of-bias assessment of randomized trials (RoB 2), traffic-light plot; Figure S3: Risk-of-bias assessment of non-randomized comparative studies (ROBINS-I), traffic-light plot; Table S1: PRISMA 2020 checklist.

## Abbreviations

The following abbreviations are used in this manuscript:

THD	transanal hemorrhoidal dearterialization
DG-HAL	Doppler-guided hemorrhoidal artery ligation
HAL-RAR	hemorrhoidal artery ligation with recto-anal repair
TST	tissue-selecting therapy
RCT	randomized controlled trial
NRS	non-randomized study
RR	risk ratio
CI	confidence interval
PI	prediction interval
RD	risk difference
RoB 2	Risk of Bias 2 tool
ROBINS-I	Risk Of Bias In Non-randomized Studies of Interventions
GRADE	Grading of Recommendations Assessment, Development and Evaluation
PRISMA	Preferred Reporting Items for Systematic Reviews and Meta-Analyses
