# Supplementary material for: Long-Term Recurrence After Transanal Hemorrhoidal Dearterialization Versus Excisional and Stapled Surgery for Hemorrhoidal Disease: A Systematic Review and Meta-Analysis of Studies with ≥24 Months of Follow-Up
Source: J Clin Med. 2026 Sep 30;15(19):7606. doi: 10.3390/jcm15197606 (PMC13665716; doi:10.3390/jcm15197606)
Supplement: Supplementary file 1 [file jcm-15-07606-s001.zip › jcm-4570001-supplementary.pdf]

SUPPLEMENTARY MATERIAL

Long-term recurrence and reintervention after transanal hemorrhoidal dearterialization versus excisional and stapled surgery: a systematic review and meta-analysis of studies with ≥24 months of follow-up. PROSPERO: CRD420261362253.

Supplementary Text S1. Full search strategies

All databases were searched from inception to August 19, 2026, with no language restriction. Searches combined intervention and comparator terms only; outcome terms were deliberately omitted. Three reviewers executed the searches independently; results were merged and deduplicated (total records identified: 932; duplicates removed: 71).

PubMed/MEDLINE

```
("hemorrhoid*"[tiab] OR Hemorrhoids[Mesh]) AND ("dearterialization"[tiab] OR
"dearterialisation"[tiab] OR "THD"[tiab] OR "hemorrhoidal artery ligation"[tiab] OR
"haemorrhoidal artery ligation"[tiab] OR "HAL"[tiab] OR "HAL-RAR"[tiab] OR
"mucopexy"[tiab] OR "anopexy"[tiab] OR "recto-anal repair"[tiab] OR "rectoanal
repair"[tiab]) AND ("hemorrhoidectomy"[tiab] OR "haemorrhoidectomy"[tiab] OR
Hemorrhoidectomy[Mesh] OR "hemorrhoidopexy"[tiab] OR "haemorrhoidopexy"[tiab] OR
"stapled"[tiab] OR "Milligan"[tiab] OR "Ferguson"[tiab] OR "LigaSure"[tiab] OR
"tissue selecting"[tiab] OR "TST"[tiab] OR randomized[tiab] OR randomised[tiab] OR
comparative[tiab] OR cohort[tiab])
```

Embase (Emtree-adapted)

```
('hemorrhoid'/exp OR hemorrhoid*:ti,ab) AND (dearterialization:ti,ab OR
dearterialisation:ti,ab OR thd:ti,ab OR 'hemorrhoidal artery ligation':ti,ab OR
'haemorrhoidal artery ligation':ti,ab OR hal:ti,ab OR 'hal-rar':ti,ab OR
mucopexy:ti,ab OR anopexy:ti,ab OR 'rectoanal repair':ti,ab) AND
('hemorrhoidectomy'/exp OR hemorrhoidectomy:ti,ab OR haemorrhoidectomy:ti,ab OR
hemorrhoidopexy:ti,ab OR haemorrhoidopexy:ti,ab OR stapled:ti,ab OR milligan:ti,ab
OR ferguson:ti,ab OR ligasure:ti,ab OR 'tissue selecting':ti,ab OR tst:ti,ab OR
randomized:ti,ab OR randomised:ti,ab OR comparative:ti,ab OR cohort:ti,ab)
```

Scopus

```
TITLE-ABS-KEY(hemorrhoid* OR haemorrhoid*) AND TITLE-ABS-KEY(dearterialization OR
dearterialisation OR thd OR "hemorrhoidal artery ligation" OR "haemorrhoidal artery
ligation" OR hal OR "hal-rar" OR mucopexy OR anopexy OR "rectoanal repair") AND
TITLE-ABS-KEY(hemorrhoidectomy OR haemorrhoidectomy OR hemorrhoidopexy OR
haemorrhoidopexy OR stapled OR milligan OR ferguson OR ligasure OR "tissue
selecting" OR tst OR randomized OR randomised OR comparative OR cohort)
```

Web of Science Core Collection

```
TS=(hemorrhoid* OR haemorrhoid*) AND TS=(dearterialization OR dearterialisation OR
THD OR "hemorrhoidal artery ligation" OR "haemorrhoidal artery ligation" OR HAL OR
"HAL-RAR" OR mucopexy OR anopexy OR "rectoanal repair") AND TS=(hemorrhoidectomy OR
haemorrhoidectomy OR hemorrhoidopexy OR haemorrhoidopexy OR stapled OR Milligan OR
Ferguson OR LigaSure OR "tissue selecting" OR TST OR randomized OR randomised OR
comparative OR cohort)
```

Cochrane CENTRAL

```
(hemorrhoid* OR haemorrhoid*):ti,ab,kw AND (dearterialization OR dearterialisation
OR THD OR "hemorrhoidal artery ligation" OR "haemorrhoidal artery ligation" OR HAL
OR "HAL-RAR" OR mucopexy OR anopexy OR "rectoanal repair"):ti,ab,kw AND
```

```
(hemorrhoidectomy OR haemorrhoidectomy OR hemorrhoidopexy OR haemorrhoidopexy OR
stapled OR Milligan OR Ferguson OR LigaSure OR "tissue selecting" OR TST):ti,ab,kw
```

Supplementary sources

ClinicalTrials.gov and the WHO ICTRP were searched for completed and ongoing trials (terms: hemorrhoid AND dearterialization/ligation); Google Scholar (first 200 records) was screened; and backward and forward citation chasing was performed on all included studies and prior systematic reviews.

Supplementary figures

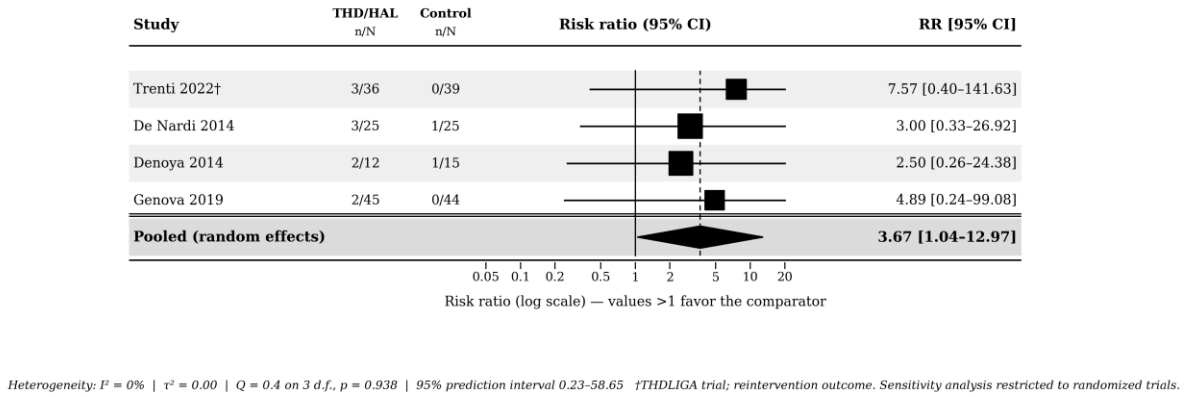

Figure S1. Sensitivity analysis restricted to randomized trials: recurrence at ≥24 months, dearterialization versus excisional hemorrhoidectomy (4 trials).

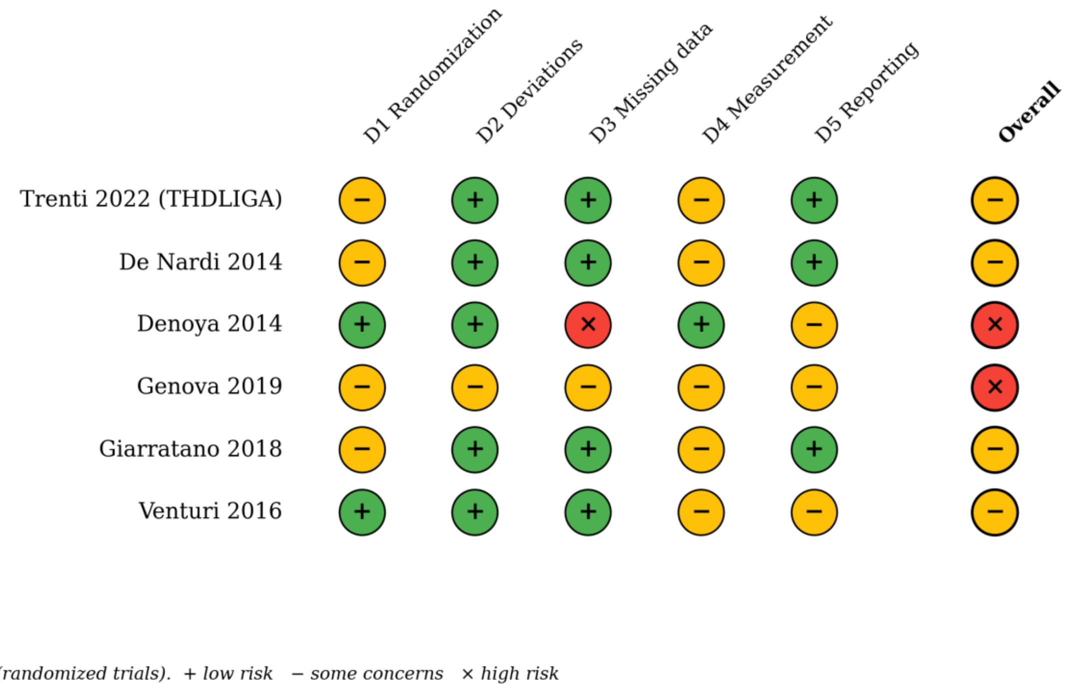

Figure S2. Risk-of-bias assessment of randomized trials (RoB 2), traffic-light plot.

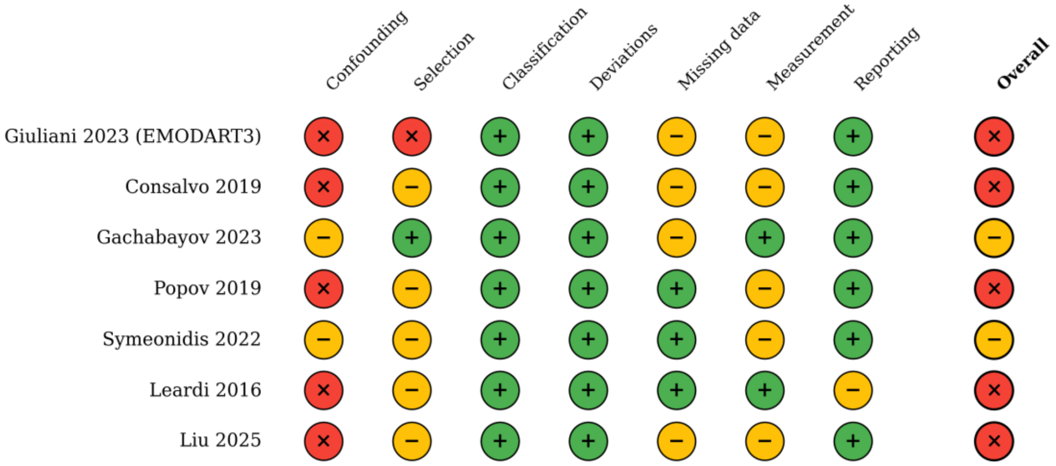

ROBINS-I (non-randomized studies). + low risk − moderate risk × serious risk

Figure S3. Risk-of-bias assessment of non-randomized comparative studies (ROBINS-I), traffic-light plot.

Table S1. PRISMA 2020 checklist

Locations refer to the numbered sections of the main manuscript (Journal of Clinical Medicine format).

Section and Topic	Item #	Checklist item	Location where item is reported
**TITLE**			
Title	1	Identify the report as a systematic review.	Title; “systematic review and meta-analysis” stated in the title
**ABSTRACT**			
Abstract	2	See the PRISMA 2020 for Abstracts checklist.	Abstract (structured: Background/Objectives, Methods, Results, Conclusions)
**INTRODUCTION**			
Rationale	3	Describe the rationale for the review in the context of existing knowledge.	Section 1, paragraph 2 (rationale; limitations of prior syntheses and recent literature)
Objectives	4	Provide an explicit statement of the objective(s) or question(s) the review addresses.	Section 1, final paragraph (objective with PICO elements)
**METHODS**			
Eligibility criteria	5	Specify the inclusion and exclusion criteria for the review and how studies were grouped for the syntheses.	Section 2.2
Information sources	6	Specify all databases, registers, websites, organisations, reference lists and other sources searched or consulted to identify studies. Specify the date when each source was last searched or consulted.	Section 2.3 (five databases, trial registries, Google Scholar, citation chasing; searched to 19 August 2026)
Search strategy	7	Present the full search strategies for all databases, registers and websites, including any filters and limits used.	Section 2.3; full per-database strategies in Supplementary Appendix S1
Selection process	8	Specify the methods used to decide whether a study met the inclusion criteria of the review, including how many reviewers screened each record and each report retrieved, whether they worked independently, and if applicable, details of automation tools used in the process.	Section 2.3 (three independent searchers; dual screening; third-reviewer adjudication)
Data collection process	9	Specify the methods used to collect data from reports, including how many reviewers collected data from each report, whether they worked independently, any processes for obtaining or confirming data from study investigators, and if applicable, details of automation tools used in the process.	Section 2.4 (duplicate extraction; author contact for missing data)
Data items	10a	List and define all outcomes for which data were sought. Specify whether all results that were compatible with each outcome domain in each study were sought (e.g. for all measures, time points, analyses), and if not, the methods used to decide which results to collect.	Section 2.2 (primary and secondary outcomes); Section 2.4
Data items	10b	List and define all other variables for which data were sought (e.g. participant and intervention characteristics, funding sources). Describe any assumptions made about any missing or unclear information.	Section 2.4 (study characteristics, intervention and comparator details, follow-up, outcome definitions)
Study risk of bias assessment	11	Specify the methods used to assess risk of bias in the included studies, including details of the tool(s) used, how many reviewers assessed each study and whether they worked independently, and if applicable, details of automation tools used in the process.	Section 2.4 (RoB 2 / ROBINS-I, two independent assessors)
Effect measures	12	Specify for each outcome the effect measure(s) (e.g. risk ratio, mean difference) used in the synthesis or presentation of results.	Section 2.5 (risk ratios with 95% CI)
Synthesis methods	13a	Describe the processes used to decide which studies were eligible for each synthesis (e.g. tabulating the study intervention characteristics and comparing against the planned groups for each synthesis (item #5)).	Section 2.5 (separate syntheses by study design and comparator class)
Synthesis methods	13b	Describe any methods required to prepare the data for presentation or synthesis, such as handling of missing summary statistics, or data conversions.	Section 2.5 (0.5 continuity correction; Peto OR and risk difference for zero-cell outcomes)
Synthesis methods	13c	Describe any methods used to tabulate or visually display results of individual studies and syntheses.	Table 1; Figures 2, 3, 5 and 6
Synthesis methods	13d	Describe any methods used to synthesize results and provide a rationale for the choice(s). If meta-analysis was performed, describe the model(s), method(s) to identify the presence and extent of statistical heterogeneity, and software package(s) used.	Section 2.5 (DerSimonian–Laird random effects; I^2, τ^2, prediction intervals)
Synthesis methods	13e	Describe any methods used to explore possible causes of heterogeneity among study results (e.g. subgroup analysis, meta-regression).	Section 2.5 (pre-specified subgroups: grade, mucopexy, follow-up stratum, recurrence definition, comparator subtype)
Synthesis methods	13f	Describe any sensitivity analyses conducted to assess robustness of the synthesized results.	Section 2.5 (≥36-month, RCT-only, low/moderate risk of bias, ≥18-month, leave-one-out)
Reporting bias assessment	14	Describe any methods used to assess risk of bias due to missing results in a synthesis (arising from reporting biases).	Section 2.5 (funnel plot and Egger test where ≥10 studies)
Certainty assessment	15	Describe any methods used to assess certainty (or confidence) in the body of evidence for an outcome.	Section 2.5 (GRADE)
**RESULTS**			
Study selection	16a	Describe the results of the search and selection process, from the number of records identified in the search to the number of studies included in the review, ideally using a flow diagram.	Section 3.1; Figure 1 (932 identified → 861 screened → 58 assessed → 13 included)
Study selection	16b	Cite studies that might appear to meet the inclusion criteria, but which were excluded, and explain why they were excluded.	Section 3.1 (Lauricella 2024 excluded from the primary analysis, reason stated); Table 1 footnote

Section and Topic	Item #	Checklist item	Location where item is reported
Study characteristics	17	Cite each included study and present its characteristics.	Table 1; Section 3.1
Risk of bias in studies	18	Present assessments of risk of bias for each included study.	Section 3.2; Table 1 (risk-of-bias column); Figures S2 and S3
Results of individual studies	19	For all outcomes, present, for each study: (a) summary statistics for each group (where appropriate) and (b) an effect estimate and its precision (e.g. confidence/credible interval), ideally using structured tables or plots.	Figures 2, 3, 5, 6 and S1 (per-study events, RR, 95% CI, weights)
Results of syntheses	20a	For each synthesis, briefly summarise the characteristics and risk of bias among contributing studies.	Sections 3.3–3.7; Table 1
Results of syntheses	20b	Present results of all statistical syntheses conducted. If meta-analysis was done, present for each the summary estimate and its precision (e.g. confidence/credible interval) and measures of statistical heterogeneity. If comparing groups, describe the direction of the effect.	Sections 3.3–3.6 (RR 1.76 [1.36–2.27] vs. excisional; RR 2.07 [0.89–4.81] vs. stapled; complications RR 0.76; late complications RR 0.19, Peto OR 0.13, RD −3.5%)
Results of syntheses	20c	Present results of all investigations of possible causes of heterogeneity among study results.	Section 3.5 (grade); Section 3.7 (recurrence definition; follow-up strata; subgroups not estimable declared)
Results of syntheses	20d	Present results of all sensitivity analyses conducted to assess the robustness of the synthesized results.	Section 3.3 (leave-one-out; RCT-only; THDLIGA exclusion); Section 3.7 (≥36-month; low/moderate risk of bias; ≥18-month not performed)
Reporting biases	21	Present assessments of risk of bias due to missing results (arising from reporting biases) for each synthesis assessed.	Section 3.7 (funnel plot and Egger test not performed: no comparison reached the pre-specified ≥10 studies)
Certainty of evidence	22	Present assessments of certainty (or confidence) in the body of evidence for each outcome assessed.	Section 3.7; Table 2 (GRADE Summary of Findings)
**DISCUSSION**			
Discussion	23a	Provide a general interpretation of the results in the context of other evidence.	Section 4, paragraphs 1–3 (interpretation against prior syntheses and recent evidence)
Discussion	23b	Discuss any limitations of the evidence included in the review.	Section 4.1 (non-randomized majority; definition heterogeneity; single-study weight; sparse late-complication events)
Discussion	23c	Discuss any limitations of the review processes used.	Section 4.1 (study-design terms in the search strategy; incomplete complication reporting)
Discussion	23d	Discuss implications of the results for practice, policy, and future research.	Section 4, Section 4.2 (future directions) and Section 5
**OTHER INFORMATION**			
Registration and protocol	24a	Provide registration information for the review, including register name and registration number, or state that the review was not registered.	Title page and Section 2.1 (PROSPERO CRD420261362253)
Registration and protocol	24b	Indicate where the review protocol can be accessed, or state that a protocol was not prepared.	Section 2.1 (registered protocol accessible at PROSPERO, CRD420261362253)
Registration and protocol	24c	Describe and explain any amendments to information provided at registration or in the protocol.	Section 2.1 (amendments preceded formal screening and are documented in the registration)
Support	25	Describe sources of financial or non-financial support for the review, and the role of the funders or sponsors in the review.	Back matter – Funding: KAU Endowment (WAQF), King Abdulaziz University, Jeddah, Saudi Arabia; technical and financial support from WAQF and the Deanship of Scientific Research (DSR)
Competing interests	26	Declare any competing interests of review authors.	Back matter – Conflicts of Interest (none declared)
Availability of data, code and other materials	27	Report which of the following are publicly available and where they can be found: template data collection forms; data extracted from included studies; data used for all analyses; analytic code; any other materials used in the review.	Back matter – Data Availability Statement (data contained within the article and Supplementary Materials; analysis code available on request)
